# Reticulate evolution in *Panicum* (Poaceae): the origin of tetraploid broomcorn millet, *P. miliaceum*

**DOI:** 10.1093/jxb/eru161

**Published:** 2014-04-10

**Authors:** Harriet V. Hunt, Farah Badakshi, Olga Romanova, Christopher J. Howe, Martin K. Jones, J. S. Pat Heslop-Harrison

**Affiliations:** ^1^McDonald Institute for Archaeological Research, University of Cambridge, Downing Street, Cambridge CB2 3ER, UK; ^2^University of Leicester, Department of Biology, Leicester LE1 7RH, UK; ^3^N.I. Vavilov Research Institute of Plant Industry, 42–44, Bolshaya Morskaya Street, 190000, St Petersburg, Russia; ^4^Department of Biochemistry, University of Cambridge, Tennis Court Road, Cambridge CB2 1QW, UK; ^5^Department of Archaeology and Anthropology, University of Cambridge, Downing Street, Cambridge CB2 3DZ, UK

**Keywords:** Crop ancestors, domestication, Panicum, hybridization, *polyploidy*, genomic *in situ* hybridization

## Abstract

*Panicum miliaceum* (broomcorn millet) is a tetraploid cereal, which was among the first domesticated crops, but is now a minor crop despite its high water use efficiency. The ancestors of this species have not been determined; we aimed to identify likely candidates within the genus, where phylogenies are poorly resolved. Nuclear and chloroplast DNA sequences from *P. miliaceum* and a range of diploid and tetraploid relatives were used to develop phylogenies of the diploid and tetraploid species. Chromosomal *in situ* hybridization with genomic DNA as a probe was used to characterize the genomes in the tetraploid *P. miliaceum* and a tetraploid accession of *P. repens*. *In situ* hybridization showed that half the chromosomes of *P. miliaceum* hybridized more strongly with labelled genomic DNA from *P. capillare*, and half with labelled DNA from *P. repens*. Genomic DNA probes differentiated two sets of 18 chromosomes in the tetraploid *P. repens.* Our phylogenetic data support the allotetraploid origin of *P. miliaceum*, with the maternal ancestor being *P. capillare* (or a close relative) and the other genome being shared with *P. repens*. Our *P. repens* accession was also an allotetraploid with two dissimilar but closely related genomes, the maternal genome being similar to *P. sumatrense*. Further collection of *Panicum* species, particularly from the Old World, is required. It is important to identify why the water-efficient *P. miliaceum* is now of minimal importance in agriculture, and it may be valuable to exploit the diversity in this species and its ancestors.

## Summary

Sequence data and genomic *in situ* hybridization identify the New World diploid *Panicum capillare* as an ancestral genome of the Old World tetraploid cereal broomcorn millet, *P. miliaceum.*

## Introduction

Identification of the wild ancestors of crops is essential for understanding their evolutionary history, including during and after domestication changes (Hammer, 1984; Vaughan *et al.*, 2007). About a third of plant species—both domesticated and wild—are polyploids (Wood *et al.*, 2009). Identification of the ancestral genome donors of polyploid crops, including *Panicum miliaceum*, can allow resynthesis to broaden the genetic basis and identify novel adaptive genotype combinations.

The grass genus *Panicum* (Panicoideae, Poaceae) is variously circumscribed (see e.g. Clayton and Renvoize, 1986, Aliscioni *et al.*, 2003). Interpreted broadly, it includes c. 500 pantropical and a few temperate species, with both C_3_ and C_4_ species. Several basic chromosome numbers have been reported, of which the most common are *x*=9 and *x*=10 (Aliscioni *et al.*, 2003). A molecular phylogeny of primarily New World species has facilitated infrageneric classification, including the proposal that the genus *Panicum* should be restricted to a group of c. 100 species (*Panicum sensu stricto*), all of which have basic chromosome number *x*=9 (Aliscioni *et al.*, 2003). The taxonomic position of many Old World species, and the relationships within the core *Panicum* clade that includes *P. miliaceum* L., remain unresolved (Triplett *et al.*, 2012).

Proso, broomcorn or common millet (*Panicum miliaceum*) is a tetraploid cereal (2*n*=4*x*=36; Hamoud *et al.*, 1994 and references therein). Its agricultural centre of origin in North China dates back to c. 10 000 BC (Hunt *et al.*, 2008; Crawford, 2009; Liu *et al.*, 2009; Lu *et al.*, 2009). It has the shortest growing season of any cereal (60–90 days) and an exceptionally low water requirement (Baltensperger, 1996; Baltensperger, 2002; Graybosch and Baltensperger, 2009). The species shows considerable morphological variation, but isozyme or microsatellite molecular marker variation is low (Warwick, 1987; Hunt *et al.*, 2011), probably reflecting the double-bottleneck of polyploidization and domestication.

*Panicum miliaceum* was cultivated widely across Eurasia in prehistory (Jones, 2004; Hunt *et al.*, 2011; Zohary *et al.*, 2012), but is a minor cereal today in terms of global economic importance, with a global production of 5 Mt, about 1% of that of wheat or rice (calculated from statistics sourced from http://faostat.fao.org/site/567/DesktopDefault.aspx?PageID=567#ancor; last accessed 13 March 2013, and http://www.fao.org/docrep/W1808E/w1808e0l.htm#annex; last accessed 13 March 2013). However, proso millet remains a locally important staple and source of food security in semi-arid regions where other cereals fail. The crop was introduced into North America in the 18th century (Dekker *et al.*, 1981) and is now grown primarily for the high-value market niche as birdseed (Graybosch and Baltensperger, 2009).

*Panicum miliaceum* is known only as a tetraploid species (2*n*=4*x*=36) and has been suggested to be an allotetraploid as it has been found to show exclusive bivalent formation at meiosis (Hamoud *et al.*, 1994). No wild tetraploid progenitor of domesticated *P. miliaceum* has been identified. Weedy forms, which may include the wild ancestor, are found across Eurasia, from Northeastern China to the Aralo-Caspian basin (Zohary *et al.*, 2012), in Central Europe (Scholz, 1983; Scholz and Mikoláš, 1991), and in North America (Bough *et al.*, 1986). Alternatively, these weedy forms may have arisen by back-mutation from the domesticate, as with fatuoid oats (Ellstrand *et al.*, 2010; Gressel, 2010).

The diploid ancestor or ancestors of tetraploid *P. miliaceum* have not been identified, and little is known about its relationship to other *Panicum* species in the *Panicum sensu stricto* clade. In the current study, *P. miliaceum*, *P. capillare* L., *P. repens* L., *P. sumatrense* Roth. ex Roem & Schult., and *P. virgatum* were investigated, based on their widespread distribution, economic significance, and their membership of the *Panicum* clade (Aliscioni *et al.*, 2003) with a base chromosome number of *x*=9. *Panicum capillare* (witchgrass) is a diploid (2*n*=2*x*=18) occurring as a weedy native to North America but naturalized in parts of central, southern, and eastern Europe (Tutin, 1980) and Asia, in the Caucasus, western Asia, the Russian Far East, and India (http://e-monocot.org/). *Panicum repens* (torpedo grass) is found in tropical and subtropical regions worldwide, including south-eastern North America, Mediterranean Europe (Tutin, 1980), the Near and Middle East, India (Moulik, 1997), China (Chen and Renvoize, 2006), and Japan. Different ploidy levels are reported for *P. repens* based on both *x*=9 and *x*=10, including diploid 2*n*=2*x*=18 (Ahsan *et al.*, 1994), tetraploid 2*n*=36 (Sinha *et al.*, 1990) and 2*n*=40 (Moulik, 1997; Aliscioni *et al.*, 2003; Freckmann and Lelong, 2003; Chen and Renvoize, 2006), and hexaploid 2*n*=6*x*=54 (Tutin, 1980), and even up to 2*n*=60 (http://www.tropicos.org/Name/25509819; last accessed 21 January 2014). The fertility of the polyploid cytotypes is unknown, and there are reports that the tetraploid weedy forms found in the US show only vegetative propagation (Majure, 2009). We do not know of any seed germplasm collections that include either a US-origin *P. repens* tetraploid, or a fertile diploid *P. repens*. *Panicum sumatrense* (little millet) is a domesticated species cultivated mainly in India, and usually reported as tetraploid with 2*n*=36 (Hamoud *et al.*, 1994; Moulik, 1997), although Chen and Renvoize (2006) report a hexaploid with 2*n*=54. *Panicum virgatum* (switchgrass) is a crop grass widespread in its native North America (Missaoui *et al.*, 2005), where it is widely planted for forage and conservation, and is a current target of development for a biofuel crop (Saski *et al.*, 2011; Triplett *et al.*, 2012). A range of chromosome numbers based on *x*=9 have been reported for *P. virgatum* (Hamoud *et al.*, 1994; Missaoui *et al.*, 2005), and a tetraploid accession (2*n*=4*x*=36; genome size 1320 Mbp; Hopkins *et al.*, 1996) is being sequenced (*Panicum virgatum* v1.1 at Jan 2014, DOE-JGI, http://www.phytozome.net/panicumvirgatum_er.php).

Phylogenetic relationships are widely studied using diverse nuclear and chloroplast DNA sequences and, for analysis of polyploids, *in situ* hybridization. 45S rDNA fragments are very widely applicable at all taxonomic levels (Hollingsworth *et al.*, 2009; China Plant BOL Group, 2011), whereas single- or low-copy genes such as the floral regulatory *FLO/LFY* gene orthologue (Bomblies and Doebley, 2005), and the endosperm starch synthesis gene granule-bound starch synthase I (*GBSSI*) have shown useful variation within groups of related taxa. Conserved chloroplast genes have also provided primer sequences of widespread taxonomic applicability in defining relationships and maternal ancestors in reticulate complexes (Hollingsworth *et al.*, 2009), although in some lineages where hybridization is involved, plastid and nuclear phylogenies may be inconsistent (Fehrer *et al.*, 2007). In hybrids and polyploids, genomic *in situ* hybridization has proved valuable to identify the genome donors and give indications about relationships between genomes or taxa such as *Nicotiana* (Patel *et al.*, 2011), *Leymus*, and *Psathyrostachys* (Orgaard and Heslop-Harrison, 1994).

The present study aimed to examine relationships between five *Panicum* species using nuclear and chloroplast gene sequences and genomic *in situ* hybridization. Genomic identity was determined in two tetraploid species (*P. miliaceum* and *P. repens*) and tested the hypothesis that the diploid species *P. capillare* is one of the ancestral genome donors of *P. miliaceum.*

## Materials and methods

### Plant material and DNA extraction

Germplasm samples were provided by the Vavilov Research Institute, St Petersburg, Russia, and by the USDA-ARS North Central Regional Plant Introduction Station, Ames, Iowa, USA. Details of the accessions used are given in Table 1. Genomic DNA was extracted from leaf tissue of single plants ground under liquid nitrogen using a modified CTAB protocol (Rogers and Bendich, 1994), or using a Qiagen DNeasy Mini Plant Kit according to the manufacturer’s protocols.

**Table 1.** *Panicum* species and accessions used in this study

Species	English name	Germplasm source	Donor ID#	Country of origin	Chromosome number
*Panicum miliaceum*	Proso	VIR	3009	Ukraine	2*n*=4*x*=36
*Panicum capillare*	Witch grass	VIR	101	France	2*n*=2*x*=18
*Panicum repens*	Torpedo grass	USDA-ARS	PI338659	Morocco	2*n*=4*x*=36
*Panicum sumatrense*	Little millet	USDA-ARS	PI197274	India	2*n*=4*x*=36

### DNA sequence analysis

*FLO/LFY-*like gene sequences spanning exon 1, intron 1, and part of exon 2 were amplified for *P. capillare, P. miliaceum, P. repens* and *P. sumatrense* using the primers 5ʹ-CCAACGACGCCTTCTCGG-3ʹ and 5ʹ-GGCACTGCTCGTACAGATGG-3ʹ (Bomblies and Doebley, 2005). Sequences were generated either from cloned plasmid DNA or colony PCR products amplified and sequenced with the *FLO/LFY* forward and reverse primers. Sequences have been submitted to GenBank (accession numbers GU444044 – GU444046, GU444048, GU444053–GU444054). Granule-bound starch synthase (*GBSSI*) sequences for the region spanning exon 2–exon 14 were generated previously for *P. miliaceum* and *P. capillare* (Hunt *et al.*, 2010; Hunt *et al.*, 2013). *GBSSI* amplifications for *P. repens* and *P. sumatrense* were performed using the primers FPSLVVC3 and Rstop3, as described previously (Hunt *et al.*, 2010); products were cloned and sequenced. Genbank accession numbers are KC477404–KC477406.

**Fig. 1.**
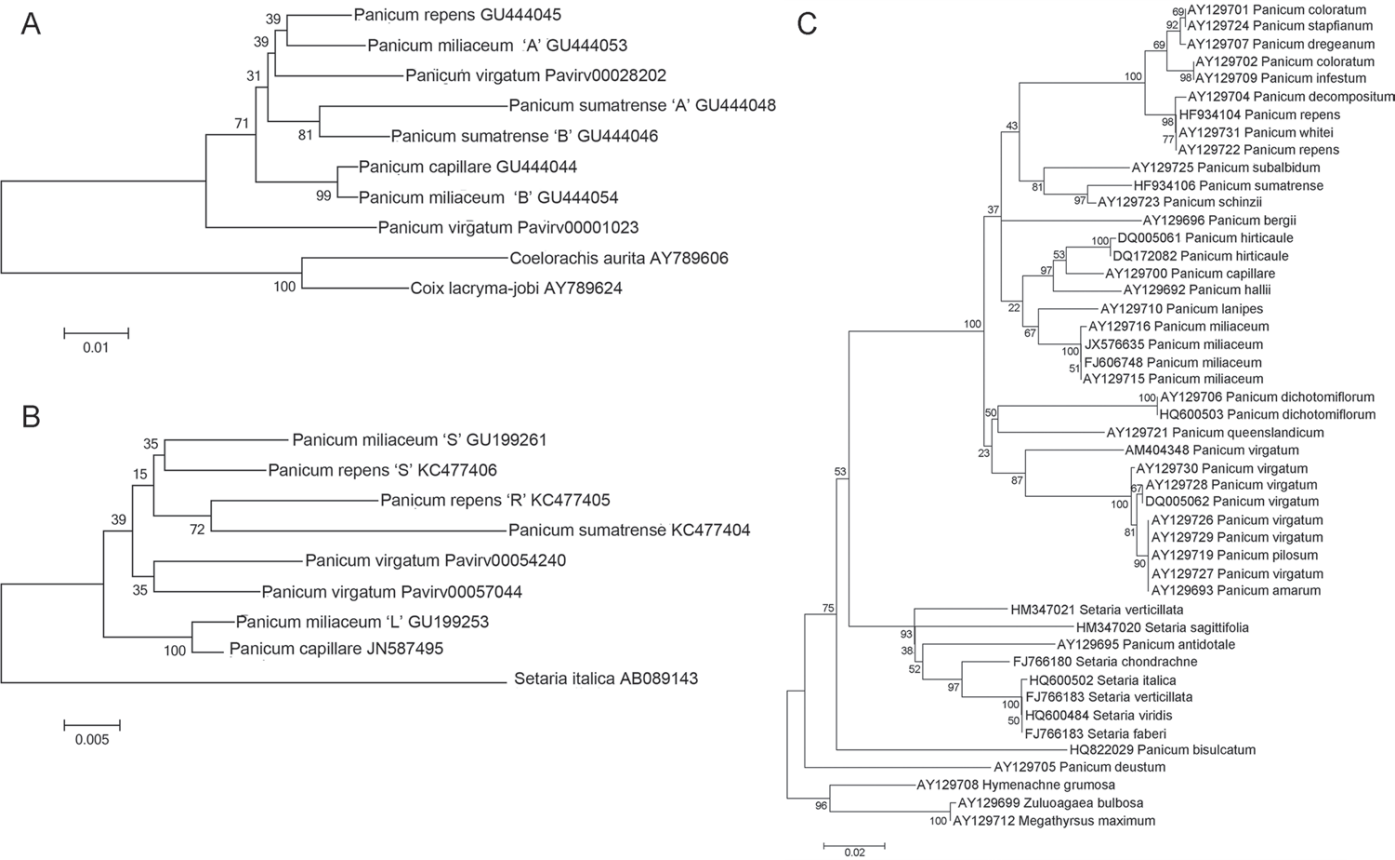
Phylogenetic trees of nuclear DNA sequences from *Panicum* and related genera, constructed in MEGA5. (A) *FLO/LFY*, Maximum Likelihood tree estimated using the Hasegawa-Kishino-Yano model with a gamma distribution parameter. Outgroups used to root the tree are *Coelorachis aurita* (GenBank accession AY789606) and *Coix lacryma-jobi* (AY789624). (B) *GBSSI*, Maximum Likelihood tree estimated using the Tamura 3-parameter model. *Setaria italica* (AB089141) was used as an outgroup. (C) ITS5: An alignment of 597bp including the 5S rRNA gene and flanking sequences from 48 *Panicum*, *Setaria*, and related species from the Genbank DNA sequence database (accession numbers shown). Only five of the many similar *P. miliaceum* sequences are shown. Maximum Likelihood tree estimated using the General Time-Reversible model with a gamma distribution parameter. The clade containing the species *Hymenachne grumosa, Zuluoagaea bulbosa*, and *Megathyrsus maximus* was used as an outgroup. Bootstrap support values based on 500 replications are shown.

Phylogenetic analysis of the *FLO/LFY* and *GBSSI* sequences included outgroups obtained from Genbank (accession numbers as in Fig. 1). Sequences for *FLO/LFY* and *GBSSI* in *P. virgatum* were obtained by BLAST searches of the *P. virgatum* genome sequence (*Panicum virgatum* v0.0, DOE-JGI, www.phytozome.net/panicumvirgatum accessed 13 March 2013). Exon sequences (following the annotation of the Genbank sequences) were aligned in MEGA5 (Tamura *et al.*, 2011), using the Muscle algorithm for codons (Edgar, 2004) and optimized by hand.

For the ITS5 nuclear sequence analysis, a region spanning about 190bp before the conserved 165bp of the 5.8S rRNA gene and 202bp after the gene, was obtained from each of 28 species of *Panicum sensu lato*, including 22 from *Panicum sensu stricto*, identified in Genbank (accession numbers as Fig. 1C) or from new sequences of PCR products amplified from *P. repens* and *P. sumatrense* using the primers 5ʹ-GGAAGGAGAAGTCGTAACAAGG-3ʹ and 5ʹ-TCCTCCG CTTATTGATATGC-3ʹ (China Plant BOL Group, 2011). Genbank accession numbers are HF934104 and HF934106. Sequences were aligned in MEGA5 (Tamura *et al.*, 2011) using the Clustal algorithm, with manual optimization: inspection suggested that Clustal performed better than the Muscle algorithm for this sequence.

DNA sequence evolution models were tested for each of the *FLO/LFY, GBSSI*, and ITS5 sequence alignments in MEGA5 (Tamura *et al.*, 2011), with complete gap deletion, which is appropriate for alignments with gaps spanning multiple nucleotides (see authors’ guide to the software). The most likely sequence evolution model (that with the lowest Bayesian Information Criterion score—the criterion recommended in the authors’ guide to the software) was used in each case to estimate the Maximum Likelihood tree. Statistical testing of the robustness of each topology was performed by bootstrap resampling of the sequence alignments, with the default program value of 500 replications. Bootstrap values for each interior branch of the original tree represent the percentage of resampled trees in which the branch is maintained.

The chloroplast DNA *matK* and *rbcL* sequences were obtained from Genbank for all available species of the genera *Panicum* (as recognized by the submitters, so generally *sensu lato*, the more distant species from *P. miliaceum* forming an outgroup) and *Setaria*. Sequences were aligned separately for each locus using Geneious R6 and manual optimization. For *matK*, a region of c. 1601bp was analysed, comprising 1542bp of the chloroplast-encoded group II intron maturase K flanked by 14 and 45bp regions from the *trnK-UUU* transfer RNA sequence. A region of 590bp within the chloroplast *rbcL* gene was analysed. FASTA alignments were converted to rdf format using Converter v.1.11 (Michael Campana, Boston, USA, pers. comm.). To visualize relationships between the chloroplast gene haplotypes, Median Joining networks (Bandelt *et al.*, 1999) were constructed in Network 4.6 (http://www.fluxus-engineering.com/sharenet.htm).

### *In situ* hybridization

Seeds were germinated on filter paper moistened with bottled drinking water (Ashbeck, Tesco) in Petri dishes for c. 4 d until roots were 8–12mm long. Before germination, husks were removed mechanically from seeds if necessary. Seedling root tips were pretreated with 8-hydroxyquinoline for 1h at room temperature and the tubes were transferred to iced water for 10h before fixation in fresh 3:1 ethanol:acetic acid. Chromosome spreads were made essentially following the methods in Schwarzacher and Heslop-Harrison (2000). Chromosome spreads were pretreated with pepsin (typically 10 μg ml^–1^, 20min at 37 °C). The 45S rDNA clone used was pTa71, and the 5S rDNA was pTa794, both originating from wheat. Genomic DNA and cloned probes were labelled with digoxigenin-11-dUTP or biotin-11-dUTP using the Invitrogen Bioprime CGH labelling kit or (for the plasmid pTa794) PCR labelling with M13 primers. *In situ* hybridization followed standard conditions. Typically, 120ng of each labelled genomic DNA probe was used for each slide, and 0.5–3 μg of unlabelled DNA from *P. miliaceum* (or, for the *P. repens* slides, *P. repens*) to increase the specificity of hybridization of the probe. Chromosome preparations were denatured at 78 °C for 7min and the final stringent washes were in 0.1 × SSC at 42 °C without formamide. Probe hybridization sites were detected with avidin conjugated to Alexa564- and anti-digoxigenin conjugated to FITC. After counterstaining with DAPI and mounting in Citifluor, images were collected with a ProgresC12 camera using exposure times of 10–40 s for FITC and Alexa564, and images were processed using Adobe Photoshop CS5 using only cropping, and functions that affected the whole image equally. Gaussian deblur functions were used where indicated.

## Results

### *FLO*/*LFY* sequence analysis

The *FLO/LFY* primers amplified a fragment around 900bp, with minor length variations both between and within *Panicum* species*. Panicum repens* and *P. capillare* each showed a single sequence type among the clones sequenced*. Panicum miliaceum* and *P. sumatrense* showed variation among clones that divided the clones into two distinct groups of sequences for each taxon, defined by the presence of particular indels and substitutions. Such variants could represent alleles on homologous chromosomes, homeologues from different parental genomes in an allopolyploid, and/or duplicated genes. Analysis of 10 accessions of *P. miliaceum* showed that the two *FLO/LFY* sequence types were present in all cases, suggesting the homologous-allele explanation is unlikely. Two sequences for *P. virgatum* (Pavirv00028202 and Pavirv00001023) were also found that showed high sequence similarity to other sequences in the dataset and are annotated as encoding FLO/LFY proteins.

Two sequence types are also found in *P. miliaceum* for *GBSSI*. We have previously established (Hunt *et al.*, 2010; Hunt *et al.*, 2013) that these represent two homeologues of *GBSSI.* As for *FLO/LFY*, a single *GBSSI* sequence type was found for *P. capillare*, and two sequence types for *P. virgatum* (Pavirv00057044 and Pavirv00054240). In contrast to the *FLO/LFY* data, two *GBSSI* sequence types for *P. repens* were found, and only a single sequence type for *P. sumatrense.*

Each distinct sequence for each species was represented in the data matrices. The aligned matrices of 10 taxa (*FLO/LFY*) and 9 taxa (*GBSSI*) were 741 and 1572bp long, respectively. The resulting maximum likelihood trees are shown in Fig. 1. Both the *FLO/LFY* and *GBSSI* trees identify two pairs of sister taxa. Firstly, the single *P. capillare* sequence for each locus forms a clade with one of the two *P. miliaceum* sequences, with >99% bootstrap support in each case (for *GBSSI*, the homeologue previously designated *GBSSI-*L; Hunt *et al.*, 2010.) Secondly, the other *P. miliaceum* sequence type (‘*GBSSI-*S’ at that locus) is sister to one of the *P. repens* sequences (the only *P. repens* sequence for *FLO/LFY*), though bootstrap support for this pairing is weak.

In the *FLO/LFY* tree (Fig. 1A), one of the two *P. miliaceum* (A), *P. repens*, and one of the two *P. virgatum* (Pavirv00028202) sequences form a clade with weak support, whereas the other *P. virgatum* (Pavirv00001023) sequence is more distantly related to the other *Panicum* sequences. The two *P. sumatrense* sequence types form a clade with relatively strong (81%) bootstrap support. In the *GBSSI* tree (Fig. 1B), the sole *P. sumatrense* sequence is sister to the second *P. repens* sequence (R).

In the ITS5 tree (Fig. 1C), *P. miliaceum* sequences and *P. lanipes* form a clade which is weakly recovered as sister to a clade containing *P. capillare* along with *P. hallii* and *P. hirticaule*, whereas *P. repens, P. virgatum* and *P. sumatrense* are dispersed on three other branches.

### Chloroplast DNA haplotype analysis

**Fig. 2.**
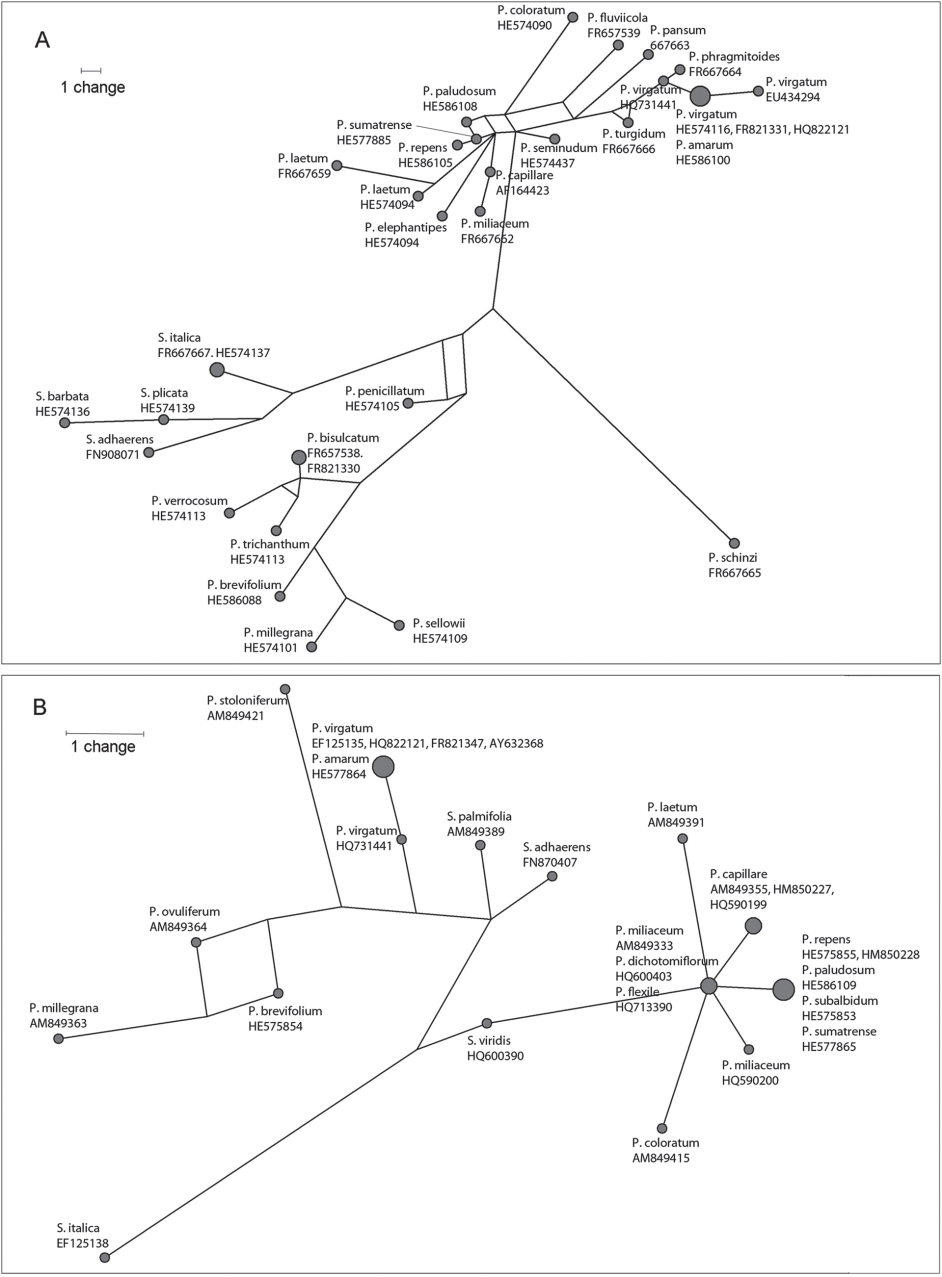
Median-joining networks (A) *matK*; (B) *rbcL*. Scale - one mutational step.

The alignment of *Panicum* and *Setaria* sequences for the *matK* locus was 1201bp in length, of which 119 sites showed variation; the *rbcL* alignment was 590bp long, of which 28 sites were variable. The set of species for which sequence data are available differs between the two loci, but in both cases includes the five *Panicum* species that are the focus of this study and the outgroup taxon *S. italica.* The median-joining networks for *matK* (Fig. 2A) and *rbcL* (Fig. 2B) both show two clusters, one including *P. miliaceum, P. capillare, P. sumatrense*, and *P. repens*, and various other species which are (Aliscioni *et al.*, 2003) or can be presumed to be *Panicum sensu stricto*. The number of nucleotide changes separating these four species is small (maximum 6). The *P. repens* and *P. sumatrense* haplotypes are identical for *rbcL* (also shared by *P. paludosum* and *P. subalbidum*), and separated by a single mutational step for *matK*. Two *P. miliaceum* haplotypes were recovered for *rbcL*, separated by one mutational step. One of these, shared with the species *P. dichotomiflorum* and *P. flexile*, was also a single base change from the *P. capillare* haplotype and from the *P. repens-P. sumatrense-P. paludosum-P. subalbidum* haplotype. In the *matK* network, the *P. miliaceum* and *P. capillare* haplotypes lie on their own branch, separated by two mutational steps, with the *P. capillare* haplotype lying two steps in from a node which is a further single step away from the *P. sumatrense* haplotype, and more distantly linked to haplotypes of several other species. Despite *P. virgatum* lying in *Panicum sensu stricto*, both *matK* and *rbcL* sequences are distant from other *Panicum sensu stricto* species (consistent with Triplett *et al.*, 2012). Indeed, for *rbcL*, *P. virgatum* and other *Panicum sensu lato* species are not monophyletic with the main *Panicum sensu stricto* clade, relative to the *Setaria* species: this may reflect the limitations of this marker for taxonomic and phylogenetic resolution.

### *In situ* hybridization

**Fig. 3.**
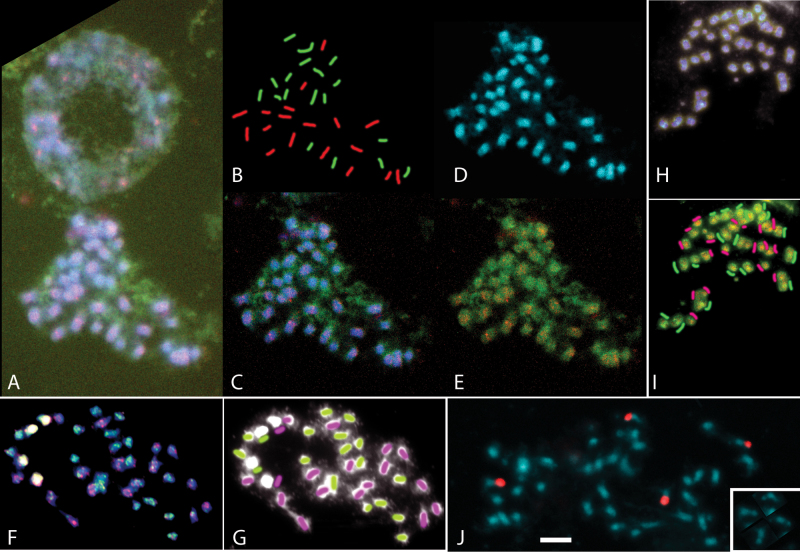
*Panicum miliaceum* (2*n*=4*x*=36) metaphases and an interphase nucleus stained blue with DAPI and hybridized *in situ* with labelled *P. repens* and *P. capillare* genomic DNA (red and green probe detection). In all metaphases, 18 chromosomes show diffuse red labelling with concentration at the centromeres and 18 are more strongly labelled with green. (A) Metaphases labelled with *P. repens* genomic DNA probe (red, generally slightly larger chromosomes) and *P. capillare* probe (green) image with contrast adjustment only; (B) overlay showing identification of chromosome ancestry; (C) Gaussian processing to show centromeric labelling of *P. repens*-origin chromosomes; (D) DAPI stained chromosomes; (E) overlay of red and green label to show stronger green labelling of 18 chromosomes with *P. capillare* probe. (F, G) A metaphase from a different slide with a different probe labelling reaction where rDNA is labelled strongly; again 18 chromosomes label green with *P. capillare* probe and 18 label red with *P. repens* probe. Four (left) label strongly with both probes at the 45S rDNA sites (white); F was processed with Gaussian functions; G, overlay showing origin of chromosomes. (H, I) A metaphase labelled with *P. repens* genomic DNA probe (green) and *P. capillare* probe (red) with (H) minimally processed image; (I) processing to emphasize differentiation and indication of chromosome origins; coloured bars indicate predominant labelling of each chromosome. (J) 45S rDNA probe (red) showing the four chromosomes carrying major rDNA loci on (inset) the satellites or secondary constrictions at the nucleolar organizing regions (NORs).

**Fig. 4.**
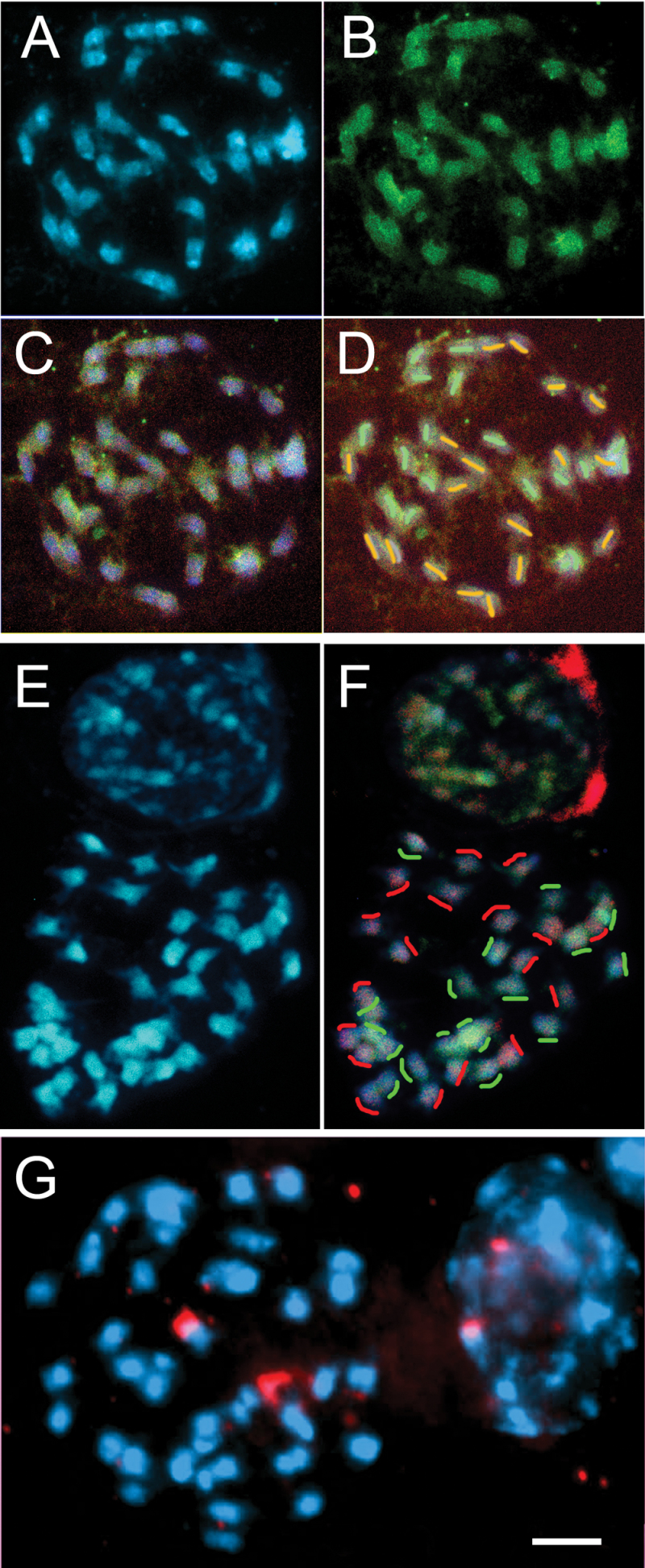
Metaphases of *P. repens* (2*n*=4*x*=36) counterstained with DAPI (blue) and probed *in situ* with genomic DNA and 45S rDNA. (A–D) Labelled with *P. capillare* genomic DNA probe (green) and *P. miliaceum* probe (red) with weak differentiation of two sets of 18 chromosomes (interpreted as those from one ancestral genome (D). (E, F) Metaphase and adjacent interphase probed with genomic DNA of *P. miliaceum* (green) and *P. sumatrense* (red)*. Panicum sumatrense* DNA (red) labels 18 chromosomes more strongly particularly in broad centromeric regions, whereas *P. miliaceum* (green) complements the *P. sumatrense* probe, with the other 18 chromosomes labelled slightly more strongly. Differentiated green and red regions are seen in the interphase nucleus (red stain precipitate present around the nucleus. (G) A metaphase and interphase hybridized with 45S rDNA (red) showing two major sites in both. Whereas differentiation of chromosome sets with the genomic DNA probe is not as strong as seen in *P. miliaceum* (Fig. 3), the result suggests *P. repens* is an allopolyploid and *P. sumatrense* is one of the ancestral genomes.

Chromosomes from *P. miliaceum* (2*n*=4*x*=36) were small, varying from 2.2–6.0 μm in length at metaphase (Fig. 3), and mostly submetacentric, with four conspicuous satellited NOR (nucleolar organizing region)-bearing chromosomes (Fig. 3J showing the 45S rDNA locations by *in situ* hybridization). There were no blocks of heterochromatin when viewed either in phase-contrast or following staining with DAPI. The *P. repens* accession (until 2012 the only one available from the NPGS/GRIN USDA-ARS germplasm collection) was tetraploid (2*n*=4*x*=36). Chromosomes were similar in morphology (Fig. 4) to *P. miliaceum*, with two NORs (Fig. 4G) labelling with 45S rDNA along most of the short arm of one pair of *P. repens* chromosomes*. Panicum capillare* (2*n*=2*x*=18, two NORs, not shown) and *P. sumatrense* (2*n*=4*x*=36, six NORs, not shown) chromosomes were generally similar in range of sizes and morphology to those of *P. miliaceum*.

To test the genome composition of the tetraploid species and the genomic relationships identified from the *FLO/LFY*, *GBSSI, ITS5*, and chloroplast DNA analyses, total genomic DNA was used as a probe to chromosomes of the tetraploid species *P. miliaceum* and *P. repens*. In metaphases of *P. miliaceum* (Fig. 3A–I) total genomic DNA of *P. repens* labelled a broad centromeric region in half (18) of the chromosomes, mostly slightly larger than the average chromosome size*. Panicum capillare* genomic DNA labelled the other half of the chromosomes with a more uniform signal, often with some exclusion from the centromere. No differentiation of *P. miliaceum* chromosomes was seen when probed with *P. sumatrense* (not shown), although technical limitations for a negative *in situ* hybridization result could not be discounted. Metaphases of *P. repens* were probed with genomic DNA of *P. miliaceum, P. capillare*, and *P. sumatrense* (Fig. 4A–F). Compared with the hybridization of genomic DNA to chromosomes of *P. miliaceum*, there was less differentiation of hybridization, and weaker signal*. Panicum capillare* DNA labelled 18 chromosomes slightly more strongly than the remaining chromosomes (Fig. 4A–D, green). The *P. sumatrense* DNA probe (red, Fig. 4E, F) labelled the arms of 18 *P. repens* chromosomes more strongly, as well as centromeric regions of most chromosomes. Genomic *P. miliaceum* DNA (Fig. 4A–D red; E, F green) probed all chromosomes with some differentiation, perhaps with slightly stronger hybridization to about half the chromosomes. Both the *P. sumatrense* and *P. miliaceum* probes sometimes labelled rDNA sites, identified in DAPI images by their weaker staining and constricted morphology.

## Discussion

Aliscioni *et al.* (2003) revised the infrageneric classifications within the genus *Panicum* using molecular and morphological data. Investigations within the section *Panicum*, particularly involving Old World species, are limited and are largely morphological. Most treatments have not taken into account the reticulate evolution, although it is clear that there is variation in ploidy within and between species, and that reticulate relationships demand further investigation (Triplett *et al.*, 2012).

The DNA sequence data (Figs 1 and 2) were used to suggest genome relationships in the group, which were then tested by *in situ* hybridization in the tetraploid taxa (Figs 4 and 5). Many of the nodes on the trees (Fig. 1) show low bootstrap support, which indicates that phylogenetic inferences are tentative. Plant molecular phylogenetics depends on markers with appropriate variation to resolve clades within the taxonomic group at the level of interest. This is particularly problematic for lower taxonomic levels (within genera and species; Dong *et al.*, 2012). Hollingsworth *et al.* (2009) have recommended the 2-locus combination of *rbcL* and *matK* (the chloroplast markers used here, Fig. 2) for species identification (barcoding). The data in Fig. 2 show that these sequences perform reasonably well in enabling species discrimination, but do not enable robust phylogenetic inferences. It is labour-intensive to develop markers *de novo* for a given study that discriminate diagnostically between the set of taxa of interest. Aliscioni *et al.* (2003), using the chloroplast *ndhF* gene, were also unable to resolve many of the species or sections within *Panicum.* Nonetheless, the trees generate useful hypotheses for testing genomic relationships, reinforced by the relatively high congruity between the loci. Chloroplast haplotype minimum spanning networks were informative for suggesting female ancestors.

The nuclear DNA sequences and *in situ* hybridization results here suggest that the exclusively tetraploid (2*n*=4*x*=36) species *Panicum miliaceum* and the tetraploid accession of *P. repens* used here are both of allotetraploid origin. It has previously been established that genomic DNA from a tetraploid species or hybrid is able to distinguish the ancestral genomes when probed to its own metaphases: in both the tetraploids *Nicotiana* × *sanderae* and *N. debneyi*, there was differentiation of the two ancestral genomes (Patel *et al.*, 2011) as we have shown here for both *P. miliaceum* and *P. repens*.

The 36 chromosomes of 4*x P. miliaceum* were labelled differentially along their length with genomic DNA from *P. repens* and *P. capillare* (Fig. 3), with some further differentiation in centromeric regions. As the signals differentiated half (18) of the chromosomes, we conclude that the probes are identifying whole chromosome sets (genomes) from the ancestral species. The hybridization and nuclear DNA results suggest that *P. capillare*, or a close relative, contributed one of the ancestral genomes. This extends the inference of genome identity between these taxa made by Triplett *et al.* (2012). The close relationship of the *P. miliaceum* and *P. capillare matK* haplotypes (Fig. 2) further suggests that *P. capillare* may have been the maternal parent, although the resolution provided by the chloroplast DNA sequence variation is not sufficient to be conclusive.

The inference of the New World native *P. capillare* as a diploid ancestor of the Old World native *P. miliaceum* is a surprising finding. The evidence that *P. capillare* is introduced in its Old World distribution seems to be largely circumstantial, and hangs on its continental (i.e. non-boreal) distribution in North America, south of the Great Lakes, its absence from Siberia and eastern Asia, and occurrence as a weed rather than in any natural habitat in Europe and Asia (Tom Cope, Kew, UK, personal communication). We can speculate that the maternal ancestor of *P. miliaceum* could have been a closely related Old World species, rather than *P. capillare* itself. However, current taxonomic knowledge of the genus is insufficient to determine which species are in the same clade as *P. capillare*. This highlights the need for germplasm collection and phylogenetic work on the Old World *Panicum sensu stricto* group and study of its biogeographical relationship to the New World group.

The similarity between the *P. capillare* and *P. miliaceum* L-homeologue *GBSSI* sequences is extremely high, not only in the exon sequence (99.3%) but also in the intron sequence (94.5%). In contrast, the intron sequences between the two *P. miliaceum GBSSI* homeologues cannot be aligned because of extensive indels (Hunt *et al.*, 2013). This further emphasises the close relationship between the *P. capillare* genome and one of the *P. miliaceum* genomes. We have previously demonstrated evidence for post-polyploidization functional changes in the *P. miliaceum GBSSI* homeologues derived from *P. capillare.* The *GBSSI* gene in *P. capillare* produces a functional protein that synthesizes endosperm amylose. In *P. miliaceum*, the wild-type gene copy encodes a protein with reduced functionality, through unknown genetic or epigenetic mechanisms (Hunt *et al.*, 2013). The other *GBSSI* homeologue in *P. miliaceum* is fully functional and determines the amylose-synthesis capacity.

The range of chromosome numbers reported in *P. repens* has led to previous speculation whether these represent an autopolyploid series or allopolyploid cytotypes (Majure, 2009). The allopolyploid origin of the tetraploid *P. repens* accession analysed here is supported by the differential labelling of two sets of chromosomes (Fig. 4), which show weak but consistent differentiation based on *x*=9. This is supported by DNA sequence (*GBSSI*) results. The data from three nuclear DNA sequences (Fig. 1) and *in situ* hybridization (Fig. 4) provide good evidence for *P. sumatrense*, or a close relative, contributing one of the two *P. repens* genomes and being the female ancestor donating the plastid sequences (Fig. 2). Both *P. capillare* and *P. miliaceum* label 18 chromosomes slightly more strongly, suggesting the other genome is more related to *P. capillare* than the other species tested (Fig. 4). The low differentiation of hybridization of the genomic DNA probes to all 36 chromosomes of *P. repens* suggests that two genomes are closer to each other than are the two *P. miliaceum* genomes (compare Fig. 3 and Fig. 4). Furthermore, only one *FLO/LFY* sequence type was found, whereas two *GBSSI* sequence types were present (Fig.1). Most tetraploid species in the Hordeeae Martinov (syn. Triticeae Dumort.) are allopolyploids, although a few have an autotetraploid origin (e.g. *Dasypyrum breviaristatum*; Galasso *et al.*, 1997), and there are autopolyploid series within individual species (e.g. *Hordeum murinum*; Taketa *et al.*, 2000).

Further collections and analysis (including of modes of reproduction) of the range of 2*x*, 4*x*, *n*=9, and *n*=10 cytotypes reported from *P. repens* worldwide are needed to determine the taxonomic integrity of the taxon; the USDA/ARS National Plant Germplasm System GRIN (USDA, http://www.ars-grin.gov/npgs/; last accessed 2010) lists no available accessions of *P. repens*, and only one of *P. capillare*, compared with 850 accessions for the wheat A genome ancestor *Triticum monococcum* ssp. *aegilopoides*. It is possible that taxonomic revision of *P. repens* may be required as with the recent revision of the model grass *Brachypodium distachyon*. Three cytotypes (2*n*=10, 2*n*=20, and 2*n*=30) were previously thought to represent an autopolyploid series within this single species. Novel cytogenetic and DNA sequence analysis showed that *B. distachyon* should be restricted to the 2*n*=10 cytotype. The 2*n*=20 cytotype is now recognised as a diverged diploid species (named *B. stacei*), and the 2*n*=30 cytotype as an allotetraploid (named *B. hybridum*) derived from *B. distachyon* and *B. stacei* (Catalán *et al.*, 2012).

For both *FLO/LFY* and *GBSSI*, two distinct *P. miliaceum* sequences were identified. For the 45S rDNA sequence fragment spanning the 5.8S rRNA gene, our sequence and 180 other *P. miliaceum* sequences from Genbank (including 175 from Y. Xu, Jilin, China) were placed on a single well-supported branch (Fig. 1C). As there are two NOR loci (4 rDNA sites), one from each genome (Fig. 3G), this provides evidence for homogenization of the ancestral DNA sequences, and replacement of all copies by a variant similar to that in *P. capillare*. Wendel *et al.* (1995) have investigated 45S rDNA homogenization in tetraploid cotton, showing the replacement of the locus from one of the *Gossypium* ancestors of several tetraploid species including *G. hirsutum* and *G. barbardense*, leaving only one rDNA variant in the tetraploid. It seems likely that the *P. miliaceum* rDNA loci, as in cotton (Wendel *et al.*, 1995), have homogenized by interlocus concerted evolution in the allopolyploid, and it is probable this involves the entire repeat.

In contrast to *P. miliaceum, P. repens* has lost whole rDNA loci (chromosomal sites), as it has only one pair compared with the three in *P. sumatrense* (Fig. 2). In *Megathyrsus maximus* (formerly in the genus *Panicum*), a diploid accession (2*n*=2*x*=16) had four 45S rDNA sites, whereas different tetraploid (2*n*=4*x*=32) accessions had 4, 6, or 8 (in a colchicine-induced tetraploid) 45S rDNA sites (Akiyama *et al.*, 2008). Similarly, in the Hordeeae (syn. Triticeae), loss of rDNA loci is a regular feature of polyploids: the tetraploid *Aegilops ventricosa* (2*n*=4*x*=28) has largely lost the rDNA sites from the *Triticum tauschii* (2*n*=2*x*=14) D-genome ancestor (Bardsley *et al.*, 1999), whereas the two B genome loci in hexaploid wheat (*T. aestivum*, 2*n*=6*x*=42) dominate the much-reduced A and D genome loci.

In many major crop species, knowledge of the wild relatives and ancestors has proved valuable for introduction of new agronomic characters by broadening the genetic base available to breeders (e.g. Tester and Langridge, 2010; Heslop-Harrison and Schwarzacher, 2012). Furthermore, diversification of the number of species in agriculture is an important contribution to future food security, development of niche markets, and increasing dietary variation. Given the worldwide need for sustainable rain-fed agriculture and increasingly limited fresh water for irrigated agriculture, there is interest in improvement and increased exploitation of proso millet (Graybosch and Baltensperger 2009). Furthermore, in suitable climates and with added nitrogen and good weed control, growth of two crops per year gives increased food security, and is associated with less soil loss during fallow periods. The short time from planting to maturity makes proso millet a good candidate to introduce into such rotations, as a catch-crop, or when establishment of another crop fails. As well as abiotic stress resistance, the *Panicum* species show resistance to biotic stresses including mildew and thrips (data not shown) although, as with most minor crops, there are few systematic studies. Finally, there are also grain quality attributes such as absence of gluten and waxy starches. Thus as a grain crop, *P. miliaceum* has a range of useful genetic properties and gene alleles which, along with those of its wild relatives, are worthy of further characterization and exploitation in breeding programmes.
